# The effect of dysmenorrhea severity and interference on reactions to experimentally-induced pain

**DOI:** 10.3389/fpain.2024.1365193

**Published:** 2024-07-23

**Authors:** S. K. Rogers, K. L. Nichols, N. Ahamadeen, M. L. Shanahan, K. L. Rand

**Affiliations:** ^1^ Department of Psychology, Indiana University Indianapolis, Indianapolis, IN, United States; ^2^ Center for Innovations in Quality Effectiveness and Safety, Michael E. DeBakey VA Medical Center, Houston, TX, United States

**Keywords:** dysmenorrhea, induced pain, expectations, QST, dysmenorrhea severity, dysmenorrhea interference

## Abstract

**Introduction:**

Dysmenorrhea is associated with increased risk of chronic pain and hyperalgesia. Menstruating individuals with dysmenorrhea are more likely to have elevated pain reactivity when experiencing experimental pain, than those without. However, no study has examined intragroup differences in reactions to experimentally induced pain for individuals with dysmenorrhea. The main aim of this study was to examine the relative roles of dysmenorrhea severity and interference in the experience of experimentally-induced pain.

**Methods:**

Participants were 120 menstruating individuals involved in a larger research study examining the influence of expectations on experimentally-induced pain. As part of the study, participants completed an online questionnaire regarding demographic and menstrual information and participated in a cold pressor task. Participants were randomized into four groups based on the manipulation of two independent variables: (1) high vs. low expectations about pain severity (pain-expectations); (2) and high vs. low expectations about one's pain tolerance (self-expectations). Participants verbally rated their pain severity throughout the cold pressor task using a 0–10 scale. Regression analyses were conducted examining the relationships between dysmenorrhea experience (i.e., average severity and interference) and cold pressor data [pain severity ratings and pain tolerance (i.e., total time in the cold pressor)], controlling for the manipulated expectations and age. Then, moderation analyses were conducted examining expectation group differences.

**Results:**

When controlling for manipulated expectations and age, dysmenorrhea severity significantly predicted initial pain severity rating (*p* = 0.022) but did not predict final pain severity rating (*p* = 0.263) or pain tolerance (*p* = 0.120). Dysmenorrhea interference did not predict initial pain severity rating (*p* = 0.106), final pain severity rating (*p* = 0.134), or pain tolerance (*p* = 0.360). A moderation analysis indicated that the relationship between dysmenorrhea severity and initial pain severity rating was not moderated by pain-expectations, *χ*^2^(1) = 0.412, *p* = 0.521.

**Discussion:**

During an experimentally-induced pain task, dysmenorrhea severity but not interference predicted initial pain severity rating, such that higher levels of dysmenorrhea severity predicted greater initial pain severity rating. This suggests individuals with more severe dysmenorrhea pain may experience greater initial sensitivity to pain and be at risk for increased sensitivity to acute pain and potentially the development of chronic pain.

## 1 Introduction

Dysmenorrhea, defined as pain associated with menstruation, is one of the most common pain conditions in menstruating individuals (1, 2). Dysmenorrhea can cause intense pain and significant life interference during the menstrual cycle. Individuals who experience dysmenorrhea often report moderate to severe levels of pain (1, 3, 4). Additionally, 38% report the pain interferes with their ability to complete daily activities (2), and 10%–30% report missing work or school due to pain (1, 2). Though dysmenorrhea is cyclical (only occurring during menstruation) and circumscribed [lasting 8–72 h (1, 5)], the effects extend outside of menstruation. Throughout the menstrual cycle, dysmenorrhea severity is associated with increased psychological distress (i.e., depressive and anxiety symptoms), relationship difficulties, reduced physical activity, and reduced sleep efficacy throughout the menstrual cycle (1, 6–8). Additionally, the presence of dysmenorrhea is associated with increased clinical and experimental pain reactivity and hyperalgesia, and increased risk of developing chronic pain conditions (1, 9–12).

Severe dysmenorrhea may also increase one's risk for central sensitization of pain. Central sensitization is a pathophysiological process resulting in altered processing of pain and sensory stimuli due to changes in the central nervous system (13). Based on validated self-report measures of central sensitization, the altered processing of pain and sensory stimuli results in hyperalgesia (i.e., increased pain sensitivity) and the development of chronic pain conditions (14). There is preliminary evidence that women with greater dysmenorrhea severity are more likely to experience central sensitization (15). This connection may explain, in part, why some individuals with dysmenorrhea experience greater pain sensitivity to induced pain (1, 9, 12, 16–18); however, these findings have been inconsistent. Previous evidence of this association has examined the presence of dysmenorrhea, rather than specific aspects of the dysmenorrhea experience. A more nuanced understanding of intragroup differences in pain sensitivity for women with dysmenorrhea may provide greater insight into the connection between dysmenorrhea and central sensitization.

Finally, expectations can play a role in dysmenorrhea and pain. There are theorized to be two distinct types of pain-related expectations: (1) pain-expectations, defined as expectations about the *severity* of the pain and (2) self-expectations, defined as expectations about *managing or tolerating* pain (19, 20). Individual's expectations about pain are direct predictors of the overall pain experience. For example, pain expectations have been demonstrated as relevant in predicting response to treatment and pain outcomes (e.g., pain severity and pain interference) both clinically and experimentally (20–23). In dysmenorrhea specifically, pain-expectations predicted dysmenorrhea severity and interference, such that higher pain-expectations predicted increased dysmenorrhea severity and interference, and self-expectations predicted dysmenorrhea interference, such that higher self-expectations predicted decreased dysmenorrhea interference (24). Though expectations play a role in both pain and dysmenorrhea, the relationship between manipulated expectations of induced pain and the pain experience in menstruating individuals has not been examined.

### 1.1 Current study

The current study seeks to examine the relationship between dysmenorrhea severity and interference and the experience of induced pain. Specifically, we examined whether dysmenorrhea severity and interference were associated with menstruating individuals’ pain severity and pain tolerance during a cold pressor induced-pain task. If a relationship between dysmenorrhea and induced pain was detected, the relationship was further examined to understand the influence of expectations on the relationship. We predicted that there would be a positive correlation between dysmenorrhea severity and participant ratings of pain severity during an induced-pain task (i.e., cold pressor). We also predicted that dysmenorrhea interference would have a negative correlation with pain tolerance on an induced pain-task.

## 2 Methods

### 2.1 Participants

This study was a secondary analysis of data from a larger research study examining the influence of expectations on pain severity and tolerance. Participants were 167 healthy adults enrolled at a large, urban, Midwestern university in the US between Fall and Spring 2022. Exclusion criteria were (a) being under the age of 18; (b) history of a heart condition, fainting or seizures, frostbite, Raynaud's phenomenon, or sickle cell disease; (c) currently pregnant; (d) have a cut or open wound on non-dominant hand; (e) taken analgesics medications in the last 4 h. Of these 167 participants 47 were excluded from the following analysis due to being male (*n* = 33) and not having a menstrual period in the past 3 months (*n* = 14), resulting in a sample of 120 menstruating individuals included in the sample.

### 2.2 Procedure

Participants provided informed consent and affirmed they met inclusion criteria. Following this, participants completed a battery of surveys, including those assessing demographic information and dysmenorrhea severity and interference.^1^

Following survey completion, participants were led to believe the surveys contained a personality test designed to assess their pain tolerance. Participants were shown a sham graphic print out from the Minnesota Multiphasic Personality Inventory (MMPI) by a research assistant (see Supplementary Figure S1).^2^ Then, participants were randomized to receive one of four “pain profiles.” Participants were not informed that these pain profiles were assigned at random. In reality, we created these “pain profiles” in order to manipulate participants pain-related expectations.

A 2 by 2 design was used to assign participants to receive either high or low pain-expectations (i.e., expectations about the severity of pain they would experience during a cold pressor task) and high or low self-expectations (i.e., expectations about how long they would be able to tolerate the pain during a cold pressor task) group. Specifically, participants were read the following prompt as the manipulation: “Have a seat here for a minute while I go check out your results from the personality measure that you just filled out…In a moment, I will have you place your non-dominant hand in this cold-water bath. For this task, you will keep your palm up and opened like this (show them). You will keep your hand in the water for as long as you can but can remove it at any time that you choose. Occasionally, I will ask for you to rate your pain using this scale (show the scale). Do you have any questions about this? … On average, people can keep their hand in the water for about 1 min. Generally, people describe this pain as a [Manipulation #1: (Low pain-expectation group: *2/10 or* High pain-expectation group: *8/10*)]. (PAUSE, flash them the personality profile) According to the results from your personality test, you will probably be able to tolerate the pain [Manipulation #2: Low self-expectation group: *much shorter or* High self-expectation group: *much longer*] than the average person…If you had to estimate, how long do you think you’ll be able to keep your hand in the water? … Go ahead and submerge your hand in the water up to your wrist and keep it there for as long as you can.”

Following the manipulation, participants were asked to estimate how long (in minutes) they would be able to keep their hand in the water. Participants then completed the cold pressor task. The cold pressor water was kept between 0°C and 2°C. Participants were asked to rate their pain on a 0–10 numeric rating scale (NRS) as soon as they placed their hand in the water, and again at 15 s, 30 s, and every subsequent 30 s until they removed their hand from the water. If participants had not removed their hand from the water after 5 min, they were instructed to do so. When participants removed their hand from the water, they were asked to give a final pain rating and place their hand in a container filled with room temperature water.

Finally, participants were provided with feedback on the actual time they kept their hand in the water compared to their estimate and were debriefed, which included information about their randomization and that they had not actually completed a personality measure. Upon completion of the study, participants were granted course credit.

### 2.3 Measures

All dysmenorrhea measures were completed using a Qualtrics online survey on a desktop computer in the research laboratory and experimental pain outcomes were collected verbally throughout the cold pressor task.

#### 2.3.1 Demographic information

Participants reported their age, sex, gender, race, ethnicity, and marital status.

#### 2.3.2 Dysmenorrhea severity

Participants were asked to rate the severity of dysmenorrhea symptoms (specifically cramping and abdominal region pain) based on how severe the symptoms were during their most recent menstrual period using a numeric rating scale from 0 (“no pain”) to 10 (“the worst pain imaginable”) scale. This is a common method for measuring dysmenorrhea severity in menstruating individuals (25, 26).^3^

#### 2.3.3 Dysmenorrhea interference

Participants were asked to rate the interference cause by their most recent dysmenorrhea symptoms (specifically cramping and abdominal region pain) on a numeric rating scale from 0 (“no activity restriction”) to 10 (“unable to do any activities”) scale. Participants were asked to rate symptoms based on how interfering the symptoms were during their most menstrual recent period. This is a common method for measuring dysmenorrhea interference in women and has been shown to be reliable and valid (27, 28).

#### 2.3.4 Initial pain severity rating

Initial pain severity rating was collected as soon as participants placed their hand in the cold pressor. Participants were shown a 0–10 NRS with 0 indicating no pain and 10 indicating the worst pain imaginable and asked to rate their pain severity using the presented scale.

#### 2.3.5 Final pain severity rating

Final pain severity rating was collected immediately after participants removed their hand form the cold pressor. Participants were shown a 0–10 NRS with 0 indicating no pain and 10 indicating the worst pain imaginable and asked to rate their pain severity using the presented scale.

#### 2.3.6 Pain tolerance

Pain tolerance was operationally defined as the time participants kept their hand in the cold pressor. Pain tolerance was measured using a manual stopwatch to record the exact time between the participant placing their hand in the cold pressor and removing their hand from the cold pressor. Pain tolerance was measured in seconds.

### 2.4 Analysis

#### 2.4.1 Linear regression analysis

We conducted six linear regression models examining dysmenorrhea as a predictor of cold pressor outcomes using SPSS (IBM Corp, Armonk, NY). Predictor variables were dysmenorrhea severity and pain interference, and outcome variables were pain tolerance (time in the water in seconds), initial pain severity rating when putting their hand in the water, and final pain severity rating after removing their hand form the water. We controlled each model for age and experimental condition using contrast coding.

#### 2.4.2 Moderation analysis

Significant linear regressions were then examined for moderation using MPlus (29). If the outcomes of initial pain severity rating and final pain severity rating were significant, pain-expectations were examined as a moderator variable. If the outcome of pain tolerance was significant, self-expectations were examined as a moderator variable, based on results of the parent study indicating that pain-expectations predicted initial and final pain severity ratings and self-expectations predicted pain tolerance (30). Dysmenorrhea severity and interference served as the predictor variables, and pain tolerance, initial pain severity rating, and final pain severity rating served as outcome variables. Pain- or self-expectations served as the moderator variable or were statistically controlled for, depending on the analysis. Each model was run for low and high expectations of the moderator variable, as determined by participant randomization. The model was run with the pathway from predictor to outcome variable freed and with the pathway from predictor to outcome variable equated. The freed and equated models were compared using a chi square difference test to determine if the moderation was significant.

## 3 Results

### 3.1 Preliminary analysis

**Figure 1:**
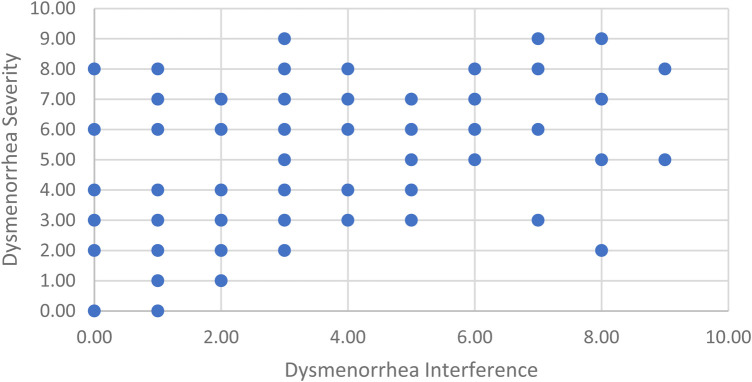
Scatterplot of dysmenorrhea severity and interference ratings.

Participants were 120 menstruating individuals, with 117 participants identifying as women and 3 participants identifying as non-binary. Participants were on average 19.16 (SD = 2.56) years of age, White (63.3%), and single (96.7%). For a full demographic breakdown of participants, see Table 1. Participants reported an average dysmenorrhea severity of 4.21 (SD = 2.39) and dysmenorrhea interference of 2.92 (SD = 2.46). Participants were able to keep their hand in the cold pressor for an average of 95.15 (SD = 91.26) seconds. They reported an average initial pain rating of 4.05 (SD = 2.32) and final pain rating of 7.44 (SD = 2.12). For a breakdown of how manipulated expectations affected pain ratings and tolerance, see Table 2. For scatter plot of participant dysmenorrhea severity and interference ratings please see Figure 1.

**Table 1:** Sample demographics.

Demographic	
Age	
Mean (SD)	19.16 (2.56)
Range	18, 38
Gender	
Women	117 (97.5%)
Non-binary	3 (2.5%)
Race—*n* (%)	
American Indian or Alaskan Native	2 (1.7%)
Asian	12 (10.0%)
Black or African American	15 (12.5%)
White	76 (63.3%)
Multiracial	7 (5.8%)
Unknown or not reported	8 (6.7%)
Ethnicity—*n* (%)	
Hispanic or Latino	30 (25.0%)
Not Hispanic or Latino	88 (73.3%)
Unknown or not reported	2 (1.7%)
Marital status—*n* (%)	
Single, never married	116 (96.7%)
Married or domestic partnership	2 (1.7%)
Divorced	1 (0.8%)
Not reported	1 (0.8%)

**Table 2:** Impact of manipulated expectations on pain ratings and tolerance.

Pain-expectations	Self-expectations					
	Low			High		
		M	SD		M	SD
Low	Initial pain	3.50	2.12	Initial pain	3.30	1.84
	Final pain	5.99	2.05	Final pain	6.83	2.62
	Pain tolerance	75.12	87.22	Pain tolerance	136.71	97.17
High	Initial pain	4.84	2.58	Initial pain	4.49	2.39
	Final pain	8.42	1.20	Final pain	8.47	1.30
	Pain tolerance	77.94	81.99	Pain tolerance	95.15	91.26

Initial pain and final pain were measured on a 0–10 scale and pain tolerance is measured in seconds.

### 3.2 Linear regression

Dysmenorrhea severity predicted initial pain severity rating (*β* = 0.22, *p* = 0.022; see Table 3) but did not predict final pain severity rating (*β* = 0.93, *p* = 0.263; see Table 4) or pain tolerance (*β* = −0.15, *p* = 0.120; see Table 5). Dysmenorrhea interference did not predict initial pain severity rating (*β* = 0.16, *p* = 0.106; see Table 6), final pain severity rating (*β* = 0.13, *p* = 0.134; see Table 7), or pain tolerance (*β* = −0.09, *p* = 0.360; see Table 8).

**Table 3:** Initial pain severity rating linear regression—dysmenorrhea severity.

	B	SE	β	*t*	*p-*value
Constant	2.39	1.64		1.46	0.147
Age	0.03	0.83	0.03	0.36	0.723
Pain-expectations	0.66	0.21	0.30	3.20	0.002
Self-expectations	−0.01	0.21	−0.01	−0.04	0.965
Dysmenorrhea severity	0.21	0.09	0.22	2.310	0.022

Regression was run controlling for age, pain-expectation group, and self-expectation group.

**Table 4:** Final pain severity rating linear regression- dysmenorrhea severity.

	B	SE	β	*t*	*p-*value
Constant	7.06	1.34		5.247	<0.001
Age	−0.01	0.07	−0.03	−0.04	0.968
Pain-expectations	1.07	0.18	0.50	5.98	<0.001
Self-expectations	0.30	0.18	0.14	1.70	0.092
Dysmenorrhea severity	0.09	0.08	0.09	1.12	0.263

Regression was run controlling for age, pain-expectation group, and self-expectation group.

**Table 5:** Actual time in water linear regression—dysmenorrhea severity.

	B	SE	β	*t*	*p-*value
Constant	205.20	64.71		3.17	0.002
Age	−4.41	3.31	−0.13	−1.33	0.186
Pain-expectations	−8.18	8.63	−0.09	−0.95	0.345
Self-expectations	21.62	8.59	0.24	2.52	0.013
Dysmenorrhea severity	−5.75	3.67	−0.15	−1.57	0.120

Regression was run controlling for age, pain-expectation group, and self-expectation group.

**Table 6:** Initial pain severity rating linear regression—dysmenorrhea interference.

	B	SE	β	*t*	*p-*value
Constant	2.85	1.70		1.68	0.097
Age	0.03	0.09	0.04	0.39	0.699
Pain-expectations	0.69	0.23	0.30	3.04	0.003
Self-expectations	−0.10	0.22	−0.04	−0.44	0.662
Dysmenorrhea interference	0.15	0.09	0.16	1.63	0.106

Regression was run controlling for age, pain-expectation group, and self-expectation group.

**Table 7:** Final pain severity rating linear regression- dysmenorrhea interference.

	B	SE	β	*t*	*p-*value
Constant	6.89	1.36		5.07	<0.001
Age	0.01	0.07	0.01	0.11	0.913
Pain-expectations	1.12	0.19	0.52	5.94	<0.001
Self-expectations	0.29	0.19	0.14	1.54	0.126
Dysmenorrhea interference	0.12	0.08	0.13	1.51	0.134

Regression was run controlling for age, pain-expectation group, and self-expectation group.

**Table 8:** Actual time in water linear regression—dysmenorrhea interference.

	B	SE	β	*t*	*p-*value
Constant	193.47	66.45		2.81	0.004
Age	−4.64	3.36	−0.14	−1.38	0.171
Pain-expectations	−12.34	9.22	0.24	2.43	0.184
Self-expectations	22.21	9.16	0.24	2.43	0.017
Dysmenorrhea interference	−3.48	3.78	−0.09	−0.92	0.360

Regression was run controlling for age, pain-expectation group, and self-expectation group.

### 3.3 Moderation

A moderation analysis was conducted on the relationship between dysmenorrhea severity and initial pain severity rating, as only this relationship was significant in the initial analysis. For the moderation analysis, the freed model was saturated. Dysmenorrhea severity did not significantly predict initial pain severity rating in the low pain-expectations group (*β* = 0.22, *p* = 0.055; see Table 9) but did significantly predict initial pain severity rating in the high pain-expectations group (*β* = 0.29, *p* = 0.029; see Table 9). The equated model showed good model fit (*χ*^2^(1) = 0.412, *p* = 0.521; RMSEA = 0.00; CFI = 1.00; SRMR = 0.02). Dysmenorrhea severity significantly predicted initial pain severity rating in the low and high pain-expectations groups (*β* = 0.27, *p* = 0.003; *β* = 0.22, *p* = 0.012 respectively; see Table 10). However, there was no significant difference between the freed and equated models (*χ*^2^(1) = 0.412, *p* = 0.521). Thus pain-expectations did not moderate the relationship between dysmenorrhea severity and initial pain severity rating.

**Table 9:** Low and high pain-expectation group model with dysmenorrhea severity paths freed.

	Standardized coefficient	Standard error	*p*-value
Low pain-expectations group			
Initial pain rating			
Dysmenorrhea severity	0.22	0.11	0.055
High pain-expectations group			
Initial pain rating			
Dysmenorrhea severity	0.29	0.13	0.029

*χ*^2^(0) = 0.00, *p* < 0.001; RMSEA = 0.00; CFI = 1.00; SRMR = 0.00. Self-expectations were controlled for in each group analysis.

**Table 10:** Low and high pain-expectation group model with dysmenorrhea severity paths equated.

	Standardized coefficient	Standard error	*p*-value
Low pain-expectations group			
Initial pain rating			
Dysmenorrhea severity	0.27	0.09	0.003
High pain-expectations group			
Initial pain rating			
Dysmenorrhea severity	0.22	0.09	0.012

*χ*^2^(1) = 0.41, *p* = 0.521; RMSEA = 0.00; CFI = 1.00; SRMR = 0.023. Self-expectations were controlled for in each group analysis.

## 4 Discussion

The overarching goal of the current study was to examine the relationship between dysmenorrhea (pain severity and pain interference) and experimentally induced pain (initial pain severity rating, final pain severity rating, and pain tolerance). In line with our hypotheses, results showed that dysmenorrhea severity predicted initial pain severity ratings. In contrast to our hypotheses, dysmenorrhea severity did not predict final pain severity ratings and dysmenorrhea interference did not predict pain tolerance. Additionally, the relationship between dysmenorrhea severity and initial pain severity rating was not moderated by pain expectations. The current study expands on previous research in several ways.

Previous research has indicated that individuals with dysmenorrhea experience hyperalgesia (10, 11), but this has not been examined beyond the mere presence of dysmenorrhea. For example, the presence of dysmenorrhea has been associated with greater pain reactivity throughout the menstrual cycle (12) and hyperalgesia during menstruation (1, 9). In the current study, we examined two aspects of the dysmenorrhea experience: severity and interference. Dysmenorrhea severity predicted initial pain severity but not final pain severity. Additionally, dysmenorrhea severity did not predict pain tolerance. These results suggest a nuanced relationship between dysmenorrhea and the experience of acute pain. Dysmenorrhea presence is associated with an increased risk of developing other chronic pain conditions (11), but this risk may be higher for individuals with greater dysmenorrhea severity. Experiencing greater acute pain severity increases the likelihood of developing chronic pain (31). Thus, individuals who experience more severe dysmenorrhea may not only be at increased risk for developing chronic pain, but also may experience increased acute pain due to increased severity of pain at the onset.

Finally, previous research indicates that pain expectations influence the pain experience. For example, Peerdeman and colleagues (20) found that manipulating expectations related to the amount of pain experienced predicted reported pain. In dysmenorrhea samples specifically, expectations of pain predicted subsequent dysmenorrhea severity and interference (24). However, results of the current study suggest expectations related to pain severity did not moderate the relationship between dysmenorrhea severity and initial pain severity ratings. This suggests that for individuals with dysmenorrhea, expectations play a role in their dysmenorrhea but not in the relationship between dysmenorrhea and the acute pain experience. Expectations not impacting the relationship between dysmenorrhea and the acute pain experience may be due to the nature of experiencing dysmenorrhea. Individuals who experience dysmenorrhea experience pain monthly, thus they may have a stronger developed sense of their abilities to experience pain, making manipulated expectations less impactful.

### 4.1 Limitations

The current study has several limitations. First, there is potential bias due to data being collected via convenience sampling of psychology undergraduates at a midwestern university. The sample is relatively young (19.16 years of age) and mostly White (63.3%). As individuals age and experience dysmenorrhea over a longer period, there may be a stronger relationship between dysmenorrhea and hyperalgesia. Additionally, previous research has indicated that central sensitization is more prevalent in White individuals with dysmenorrhea (32). Thus, the fact that the present sample comprised mostly White participants may have resulted in a stronger observed relationship between dysmenorrhea and induced pain severity. Additionally due to the modest-sized sample and all analyses determined *a priori*, we decided not to use a Bonferroni correction.

Second, information on non-dysmenorrhea pain, contraception use, and menstrual cycle phase was not collected. If individuals experience non-dysmenorrhea related chronic pain, this pain may act along the same central sensitization pathways (14), such that the non-dysmenorrhea pain increases sensitization and increase hyperalgesia. This may result in overestimation of the relationship the dysmenorrhea and induced pain. If individuals were taking hormonal contraceptives (combined oral-contractive, IUDs, and implants), their induced pain experience may have been altered because exogeneous hormones can influence pain sensitivity and non-menstrual related pain (33–36). Additionally, women may use contraceptives to help with their dysmenorrhea. If this reduces their dysmenorrhea severity, they will report a lower dysmenorrhea severity rating, which may then result in an underrepresentation of the relationship between dysmenorrhea severity and induced pain. Previous research has indicated there may be differences in pain sensitivity and tolerance across the menstrual cycle (17, 37). This may result in an underestimation of the relationship between dysmenorrhea and induced pain, given that hyperalgesia and decreased pain tolerance are greater during menstruation and follicular phases (17, 37).

Third, it was not possible to keep the cold pressor water at a constant temperature. Instead, the water temperature was maintained between 0° and 2°C. Temperature fluctuations may have introduced random variability in pain severity ratings and tolerance, making it more difficult to detect significant relationships.

### 4.2 Implications

Future research should examine how different aspects of the dysmenorrhea experience influence pain perception. Examining the influence of the menstrual phase in addition to dysmenorrhea severity could provide important information on development of chronic pain, depending on the menstrual phase when the pain experience begins. Understanding if pain experiences in specific menstrual phases impact the development of chronic pain could provide insight into individuals who may need additional treatment to reduce the likelihood of acute pain transitioning to chronic pain. Additionally, future research should examine how the complexities of dysmenorrhea influence its relationship with pain sensitivity. The symptoms of dysmenorrhea are diverse, including cramps, lower abdominal pain, pain radiating into the hips and thighs, fatigue, nausea, diarrhea, and headache (38). Certain symptoms of dysmenorrhea may affect pain sensitivity more than others. Understanding how different symptoms influence pain sensitivity would provide additional information on targeting specific symptoms of dysmenorrhea to reduce the long-term impacts of dysmenorrhea (i.e., chronic pain).

## 5 Conclusion

Dysmenorrhea is the most common pain condition in menstruating individuals. The sequelae of dysmenorrhea are far reaching, including hyperalgesia and development of chronic pain conditions. The current study expands on the knowledge of the relationship between dysmenorrhea and hyperalgesia by providing evidence that dysmenorrhea severity is predictive of initial pain sensitivity. This suggests individuals who experience more severe dysmenorrhea pain may be at increased risk for experiencing more severe acute pain.

## Data Availability

The raw data supporting the conclusions of this article will be made available by the authors, without undue reservation.

## Footnotes

^1^The battery of surveys included a number of other psychological measures, such as the Adult Hope Scale, the Life Orientation Test-Revised, Generalized Anxiety Disorder-7, Brief Cope Scale, the Positive and Negative Affect Scale, and the NEO Personality Inventory-Revised.

^2^Participants did not actually complete a personality test, or any portion of the MMPI, rather they were led to believe that the battery of surveys they completed were a personality test.

^3^There are many definitions of dysmenorrhea severity, specifically with conflict among researchers on what level of pain is considered “normal” and level of pain is considered “severe.” For the purpose of this study, we did not differentiate between “mild,” “moderate,” or “severe” dysmenorrhea, but rather considered all menstruating individuals and considered any pain rating 1 or greater as experiencing dysmenorrhea.
