# Supplementary figures and images for: The effect of dysmenorrhea severity and interference on reactions to experimentally-induced pain
Source: Front Pain Res (Lausanne). 2024 Jul 23;5:1365193. doi: 10.3389/fpain.2024.1365193 (PMC11300429; doi:10.3389/fpain.2024.1365193)

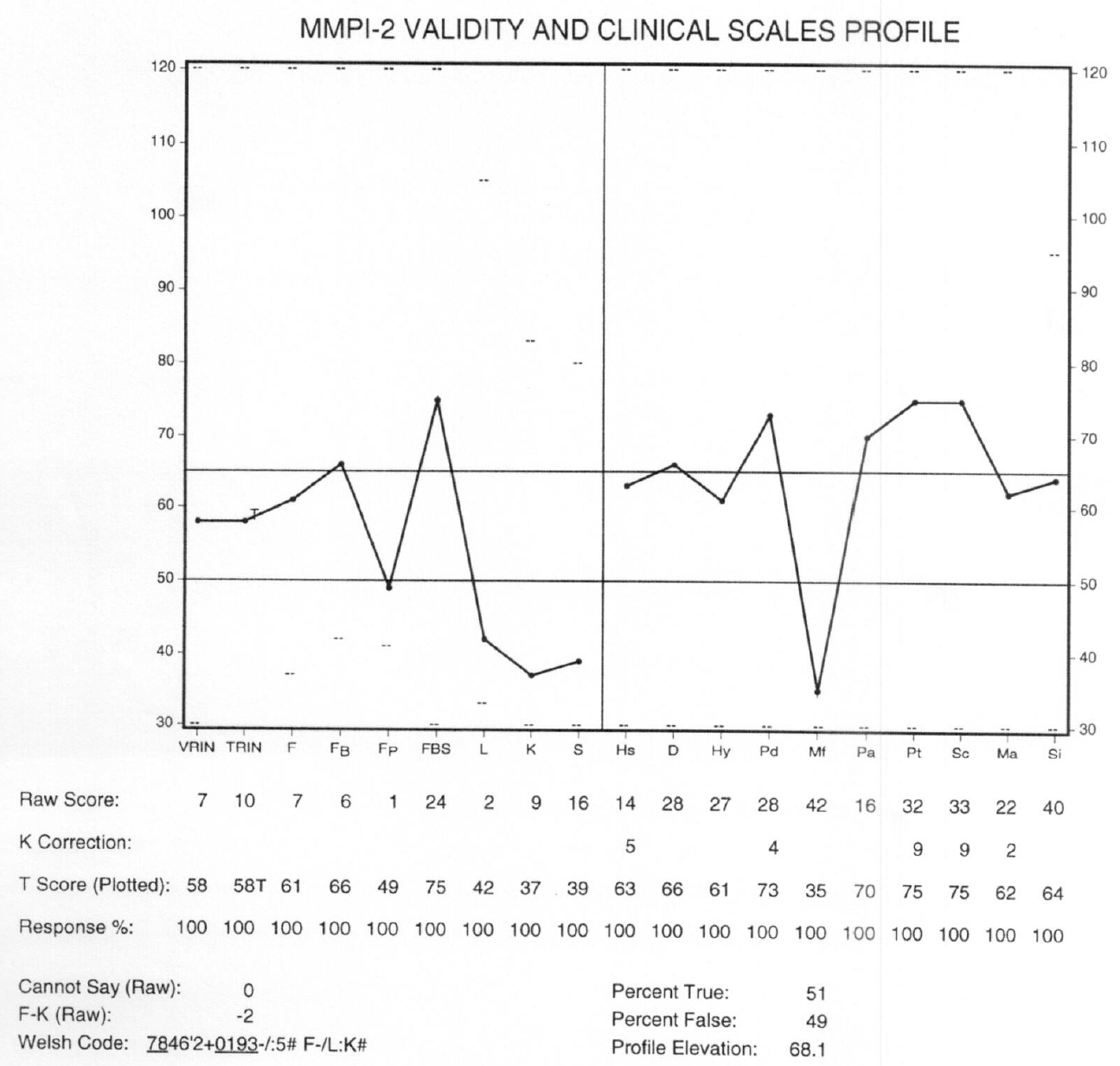

Supplement: Supplementary file 1 [file Datasheet1.docx]
